# Performance and Physician Experience of INGEVITY+ Active Fixation Leads: Prospective INGEVITY+ Lead Clinical Study in Korea

**DOI:** 10.1155/2024/2172306

**Published:** 2024-01-11

**Authors:** Boyoung Joung, Myung Hwan Bae, Il-Young Oh, Hyung-Seob Park, Jaemin Shim, Min Soo Cho, Jung Myung Lee, Eue-Keun Choi, Young Soo Lee

**Affiliations:** ^1^Yonsei University Health System, Seoul, Republic of Korea; ^2^Kyungpook National University Hospital, Daegu, Republic of Korea; ^3^Seoul National University Bundang Hospital, Soengnam-Si, Republic of Korea; ^4^Keimyung University Hospital, Daegu, Republic of Korea; ^5^Korea University Hospital, Seoul, Republic of Korea; ^6^University of Ulsan College of Medicine, Seoul, Republic of Korea; ^7^Kyung Hee University Hospital, Seoul, Republic of Korea; ^8^Seoul National University Hospital, Seoul, Republic of Korea; ^9^Daegu Catholic University Medical Center, Daegu, Republic of Korea

## Abstract

**Background:**

Boston Scientific INGEVITY+ pacing lead (Boston Scientific, Marlborough, MA, USA) has been upgraded to INGEVITY. The performance of the INGEVITY+ pacing lead has not yet been reported. This study aimed to evaluate the short- and long-term safety, effectiveness, and handling experience of INGEVITY+ leads.

**Methods:**

Consecutive patients were included from 9 institutions in Korea, where 400 leads (200 right ventricular active fixation leads and 200 right atrial active fixation leads) were implanted or attempted in 200 subjects.

**Results:**

During the implantation, only one patient required a lead change because of lead screw failure. The handling questionnaires of the lead received very positive feedback with 88% of operators agreeing that it is easy for leads to pass through small vessels or vessels with multiple leads. At the 3-month follow-up, 95.7% of RA leads and 99.5% of RV leads had pacing thresholds less than 1.5 V. A total of 92.4% of atrial leads had amplitudes greater than 1.5 mV, and 96.5% of ventricular leads had sensing amplitudes greater than 5 mV at 3 months. A total of 99.8% had impedances between 300 and 1,300 ohms. The lead-related complication-free rate for all leads during follow-up was 100%, and the overall rates of lead dislodgment, perforation, and pericardial effusion were all 0.0%.

**Conclusions:**

The INGEVITY+ pacing lead exhibited exceptional clinical performance, with a high complication-free rate throughout the 3-month follow-up period. In addition, the lead displayed excellent electrical characteristics, and the lead-handling experience was reported to be very good.

## 1. Introduction

Intracardiac leads play a vital role in transvenous pacemaker systems, serving as an insulated electrical connection between the implantable pulse generator (IPG) and the cardiac tissue. These leads face myriad challenges, including enduring biodegradation within the body's environment, withstanding repetitive flexural cycles of the heart, and handling compressive and tensile forces in the extravascular space. The design requirements for these leads encompass various aspects including ease of implant handling, fluoroscopic visualization, lead body diameter, durability to outlast multiple IPG replacements, low-energy cardiac tissue stimulation, reliable sensing of intrinsic cardiac activity, and considerations for future lead extraction.

The use of magnetic resonance imaging (MRI) for diagnostic purposes is rapidly increasing in many fields (like the brain, spinal cord, and musculoskeletal system). About 50–70% of patients with cardiac implantable electronic devices are estimated to have an indication for MRI over the lifetime of the device (1) [1]. The incorporation of MRI conditional lead design offers an added safety benefit, considering the increasing preference for MRI scans in many fields. In the past, MRI scanning was contraindicated for patients with implanted cardiac devices [2, 3], but recently, MRI scans have been safely performed in certain patients [4–9]. Despite these advancements, concerns persist regarding the potential adverse effects of MRI scanners on pacemaker function, such as tissue heating at the lead tip, leading to capture failure or induced arrhythmias due to unintended cardiac stimulation [10, 11]. Therefore, the MRI lead was modified to reduce radiofrequency lead tip heating. These modifications resulted in a larger diameter and greater stiffness of the leads compared with the conventional non-MRI pacing lead. Consequently, the Medtronic 5086 lead is reportedly associated with increased cardiac perforation and lead dislodgment [12–14].

The Boston Scientific INGEVITY+ pacing lead (Boston Scientific, Marlborough, MA, USA) was upgraded to the INGEVITY lead. The clinical performance of the INGEVITY lead has demonstrated a high lead-related complication-free rate over 12 months of follow-up and excellent electrical characteristics. However, the performance of the INGEVITY+ pacing lead has not yet been reported. This study aimed to evaluate the short- and long-term safety, effectiveness, and handling experience of INGEVITY+ pacing leads.

## 2. Methods

### 2.1. Device Characteristics

**Figure 1:**
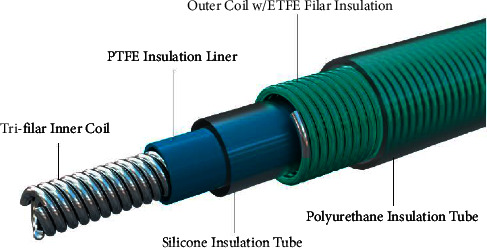
INGEVITY+ pacing lead design. INGEVITY+ lead has three layers of insulation between conductors and a polyurethane lead body. The trifilar inner coil design provides consistent, low, and repeatable turn counts when extending and retracting the helix2.

The INGEVITY+ pacing leads were 6F (2.0 mm) steroid-eluting endocardial pace/sense leads designed for permanent implantation in atrial or ventricular applications. These leads use an active fixation mechanism that employs an extendable or retractable helix for secure placement. INGEVITY+ was built on the established INGEVITY platform and incorporates specific design features for MRI conditional safety. The leads had three layers of insulation between the conductors and the polyurethane lead body to ensure optimal electrical performance and safety. The inner coil design of the leads is trifilar, providing consistent, low, and repeatable turn counts during helix extension and retraction. These leads incorporate design aspects for MRI conditional safety. The inner coil of the MRI lead was modified to have a higher inductance to prevent heating during MRI scanning, which was achieved through the unifilar design of the inner coils of the INGEVITY leads [13, 15, 16]. However, because torque transfer decreased in a unifilar inner coil, the INGEVITY+ leads were upgraded to a trifilar coil design. These leads feature an IS-1 bipolar connector for seamless integration with the pacing systems. The tip of the lead was designed with flexibility in mind, incorporating an iridium oxide (IROX™) coating on the tip of the electrode to improve the electrical performance and lead longevity. The lead design is illustrated in Figure 1. This study was approved by the Institutional Review Board of Severance Hospital (1-2021-0011), and all patients provided written informed consent.

### 2.2. Clinical Evaluation

This study evaluated the safety and effectiveness of INGEVITY+ pacemaker leads over a 3-month follow-up period. These leads were implanted in the right atrial (RA) and/or right ventricular (RV) region as part of a single chamber (SC) or dual chamber (DC) pacemaker. Patients eligible for the study had Class I or Class II indications for device implantation as per the reference guidelines.

For endpoint analyses, only leads that were implanted or attempted last during the initial implantation procedure in each chamber were considered. The safety evaluation of INGEVITY+ leads focused on the complication-free rate (CFR) related to leads from the time of lead implantation until the 3-month follow-up. CFR was determined based on complications specifically related to INGEVITY+ lead. Complications related to the leads include permanent loss of pacing therapy, injury, invasive intervention, or death. These are based on the AdvaMed document, “Industry Guidance for Uniform Reporting of Clinical Performance of Cardiac Rhythm Management Pulse Generators and Leads,” which sets standards for lead performance reporting and specifically addressed the reporting of active registry performance data.

Clinical effectiveness was assessed by evaluating the sensing and pacing performance at the 3-month postimplantation mark. The primary effectiveness endpoint involved measuring bipolar pacing thresholds, which refers to the minimum electrical stimulation required to consistently initiate cardiac depolarization. This measurement was taken in volts (V) using a 0.5-ms pulse. In addition to the pacing threshold measurement, other lead electrical performance parameters were assessed at different time points, including predischarge and 3-month follow-ups.

All lead deficiencies were documented and reported to the Institutional Review Board. The handling experiences of the implanting physicians were collected to gain insights into their experiences with the new lead. Specific aspects of interest include the radiopacity of the active fixation lead helix, which aids in confirming the full extension of the helix during implantation. The overall handling of the lead and other related questions were part of the feedback assessment.

### 2.3. Statistical Methods

Descriptive statistics (mean ± SD) were reported for lead electrical data. Comparisons between groups were performed using a 2-sample *t*-test or Fisher's exact test. The 95% lower pointwise confidence limit of the lead-related CFR was determined using the log-log methodology. These values were compared with the predefined performance goals, which were set at 91.4% for the safety endpoint. An *α* level of 0.05 was used for each analysis. Data assembly and statistical analyses were performed using SPSS version 22 (IBM Inc., Armonk, NY, USA).

## 3. Results

### 3.1. Patients

We enrolled 200 subjects from nine centers in Korea from June 2021 to September 2022. All enrolled patients underwent implantation or attempted implantation with an INGEVITY+ lead, and all patients in this group received the lead. The lead implantation procedure involved placement of both atrial and ventricular INGEVITY+ leads. Specifically, 200 RV and 200 RA active fixation leads were implanted in the study population.

All patients in this study required pacemaker implantation as part of their medical treatment. The patient demographic characteristics are presented in Table 1. Notably, 43.5% of the enrolled patients were men, while 56.5% were women. The mean age of the patients was 70.2 ± 9.1 years, with an age range spanning from 27.0 to 86.0 years. The indications for pacemaker implantation were sinus node dysfunction (40.5%) and atrioventricular (AV) block (39.0%).

**Figure 2:**
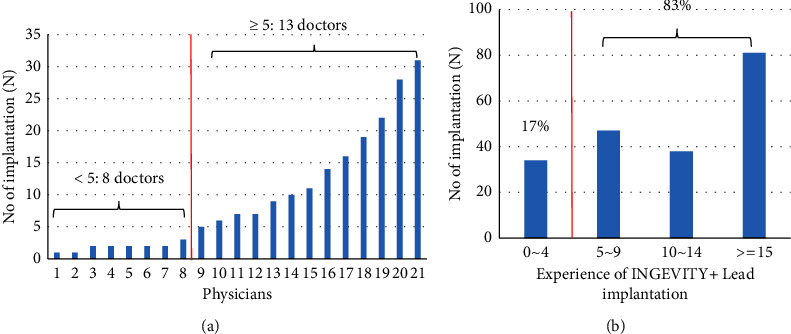
Number of implantation according to physicians (a) and the experience of physician (b).

Figure 2 shows the number of INGEVITY+ lead implantation according to physicians and the experience of physician. Most implantations (83%) were performed by physicians with INGEVITY+ lead implantation more than four (Figure 2).

### 3.2. Follow-Up and Complication-Free Rate (CFR) Related with Leads

From enrollment to the 3-month follow-up, all subjects survived and remained in the study. During the implantation procedure, one patient required a lead change because of lead screw failure.

The INGEVITY+ study successfully achieved predefined safety endpoints, and the lead-related CFR was 100% at three months. No instances of lead dislodgment were observed in any participant, resulting in an overall dislodgment rate of 0%. Additionally, the study recorded no occurrences of lead perforation (0.0%) or pericardial effusion (0.0%).

### 3.3. Electrical Measurements

#### 3.3.1. Pacing Threshold

**Figure 3:**
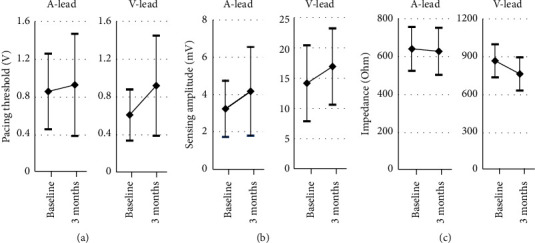
The change of lead profiles. (a) Pacing threshold, (b) sensing amplitude, and (c) impedance. A-lead indicates atrial lead and V-lead indicates ventricular lead.

Throughout the 3 months, the pacing threshold of INGEVITY+ leads remained consistently low and stable. The mean pacing threshold for active fixation in the RV was 0.92 ± 0.53 V, whereas that in the RA was 0.93 ± 0.54 V. A total of 95.7% of the RA leads and 99.5% of the RV leads exhibited pacing thresholds less than 1.5 V (Figure 3(a)).

#### 3.3.2. Sensing Amplitude

At the 3-month evaluation, the mean sensed amplitude of the INGEVITY+ leads in the RA was recorded as 4.2 ± 2.4 mV. For leads in the RV, the mean sensed amplitude was 16.9 ± 6.3 mV. Remarkably, 92.4% of the RA leads exhibited amplitudes >1.5 mV, whereas 96.5% of the RV leads exhibited amplitudes >5 mV (Figure 3(b)).

#### 3.3.3. Pacing Impedance

The mean pacing impedance measured at 3 months was 628.0 ± 123.7 ohms for RA and 765.2 ± 131.1 ohms for RV. The ventricular leads showed a decreasing impedance trend from the time of implantation to the 3-month follow-up (*p* < 0.001). In contrast, the atrial leads exhibited consistent impedances between the time of implantation and the 3-month evaluation (*p*=0.219). The impedances were between 300 and 1,300 ohms in 99.8% of leads (Figure 3(c)).

### 3.4. Handling Experiences

**Figure 4:**
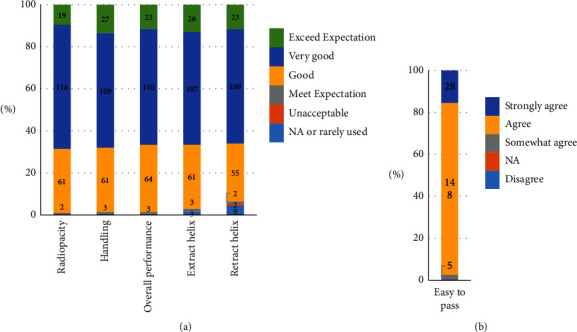
Lead-handling questionnaire. (a) The radiopacity quality, handling, overall performance, extract, and retract helix. (b) Easy to pass.

In the lead-handling questionnaires, the radiopacity quality of the extendable/retractable helix markers, as well as the handling and maneuverability of the stylet and lead, were rated as “very good” or exceeded expectations in 68.5% and 68% of cases, respectively. Regarding the overall handling performance of the leads, 66.5% of the respondents graded it as “very good” or “excellent,” while 32.0% considered it as “good,” and 1.5% found it to meet their expectations. Furthermore, a significant majority (88%) of operators agreed or strongly agreed that the leads were easy to navigate through small vessels or vessels with multiple leads (Figure 4).

## 4. Discussion

### 4.1. Safety and Effectiveness

This study provides compelling evidence of the safety and effectiveness of the INGEVITY pacemaker lead, surpassing predefined endpoints. The lead demonstrated a remarkable CFR of 97.9%, which compares favorably with other leads specifically designed for MRI environments (96.3–97.0%) and leads with longer historical usage in the field (97.3–97.9%) [13, 14, 17–19].

To achieve a higher inductance, the inner coil was reduced from a multifilar design in the MRI-noncompatible model, leading to a 1-2 filar design [13, 15]. The two-filar inner conductor coil design of the Medtronic 5086 lead increased the inductance, thereby reducing lead tip heating caused by radiofrequency (RF) energy. It has a 7F diameter, which requires an 8F introducer. However, its tip stiffness, which can be an indicator of lead perforation, is greater than that of the Medtronics 5076 model [13, 15]. Therefore, the Medtronic 5086 lead has been reported to be associated with increased cardiac perforation, tamponade, death, and lead dislodgment [12–14]. Delayed lead perforation may be associated with leads featuring a decreased diameter, resulting in higher force per unit area [19, 20].

In the case of the INGEVITY and INGEVITY+ studies, which evaluated the 6-French lead design, no increase in the perforation rate was observed. Moreover, the INGEVITY+ data did not indicate a higher incidence of lead-related complications. However, it is usually recommended that a softer stylet be used when siting the MRI leads in an apical position and that the stylet is not fully seated when the lead is positioned against the myocardium. Another major difference between the leads is the number of rotations required to fully deploy the helix in the myocardium. To facilitate the transfer of torque and prevent sudden exit of the helix, manufacturers advise that the proximal portion of the lead should be maintained in a straightened position and that one turn per second be applied. Fluoroscopy is the only reliable method for confirming extension of the helix. The movement of the radiographic markers at the lead tip indicates that the helix has been successfully deployed [13, 15].

Torque transfer was lower in a unifilar inner coil than in a similarly designed multifilar coil [12, 13]. In handling experience, INGEVITY+ lead-handling experience was reported to be very good.

### 4.2. Study Limitations

This study was designed as an observational, single-arm investigation, thus limiting direct comparisons with historical control groups. This study included high-volume pacemaker centers with experienced operators. The annual pacemaker implantation volume was inversely related to early surgical complications and early lead dislocations. Therefore, the outcomes observed in this study may differ in real-world settings, with fewer experienced operators and lower overall procedure volumes. These factors may introduce potential limitations to the generalizability of the findings to diverse clinical environments [12, 21].

## 5. Conclusion

The study findings unequivocally established the safety and effectiveness of the INGEVITY+ pacemaker lead, which exceeded the predefined endpoints. Continuous surveillance will be maintained to ensure the ongoing assessment of lead durability and long-term performance.

## Figures and Tables

**Table 1:** Baseline characteristics of patients.

Characteristics	Implanted or attempted patients
Age at implant (years)	70.2 ± 9.1
Male	87 (43.5)
BMI, kg/m^2^	24.2 ± 3.9
Height, cm	159.3 ± 14.8
Weight, kg	62.4 ± 12.7
Smoking, current	22 (11.0)
Drinking, current	21 (10.5)
Indication of PM implantation (*N* (%))	
Sinus node dysfunction	81 (40.5)
Atrioventricular block	78 (39.0)
Both	34 (17.0)
Others	7 (3.5)
Associated diseases and risk factors (*N* (%))	
Hypertension	126 (63.0)
Diabetes	61 (30.7)
Myocardial infarction	12 (6.1)
Valvular heart disease	11 (5.6)
Heart failure	14 (7.1)
Peripheral artery occlusive disease	9 (4.5)
Cerebrovascular disease	18 (9.0)
Dyslipidemia	94 (47.2)

Values are presented as mean ± standard deviation or number (%). BMI, body mass index; PM, pacemaker.

## Data Availability

The data that support the findings of this study are available from the corresponding author upon reasonable request.
